# The Involvement of Endogenous Neural Oscillations in the Processing of Rhythmic Input: More Than a Regular Repetition of Evoked Neural Responses

**DOI:** 10.3389/fnins.2018.00095

**Published:** 2018-03-07

**Authors:** Benedikt Zoefel, Sanne ten Oever, Alexander T. Sack

**Affiliations:** ^1^MRC Cognition and Brain Sciences Unit, University of Cambridge, Cambridge, United Kingdom; ^2^Department of Cognitive Neuroscience, Faculty of Psychology and Neuroscience, Maastricht University, Maastricht, Netherlands

**Keywords:** entrainment, phase, ERP, evoked response, power, oscillation, endogenous

## Abstract

It is undisputed that presenting a rhythmic stimulus leads to a measurable brain response that follows the rhythmic structure of this stimulus. What is still debated, however, is the question whether this brain response exclusively reflects a regular repetition of evoked responses, or whether it also includes entrained oscillatory activity. Here we systematically present evidence in favor of an involvement of entrained neural oscillations in the processing of rhythmic input while critically pointing out which questions still need to be addressed before this evidence could be considered conclusive. In this context, we also explicitly discuss the potential functional role of such entrained oscillations, suggesting that these stimulus-aligned oscillations reflect, and serve as, predictive processes, an idea often only implicitly assumed in the literature.

## Introduction

If a rhythmic stimulus is presented, a brain response can be measured that follows the temporal structure of the stimulus and is therefore rhythmic—or oscillatory—as well. This phenomenon is often assumed to reflect a synchronization between neural oscillations and stimulus rhythm (often termed *neural entrainment*) and has experienced a tremendous increase in popularity over recent years (Peelle and Davis, 2012; Zion Golumbic et al., 2013; Calderone et al., 2014; Wilson and Cook, 2016; Zoefel and VanRullen, 2017). The arguably most attractive idea behind the functionality of aligning oscillatory activity to a rhythmic structure is that it reflects a predictive process: any rhythmic input can, by definition, be predicted. Together with the fact that neuronal oscillations reflect rhythmic changes between low–and high–excitability phases (Buzsáki and Draguhn, 2004), it seems to be a reasonable assumption that the brain tries to selectively amplify important input by aligning the high-excitability oscillatory phase with the predicted timing of those expected events (Schroeder and Lakatos, 2009). Indeed, an *active* (i.e., initiated by the brain) synchronization between *endogenous* neural oscillations^1^ and a rhythmic stimulus is central for many theories across fields of research, ranging from attentional selection (Lakatos et al., 2008) or sensorimotor synchronization (Merker et al., 2009) to the parsing of speech (Giraud and Poeppel, 2012) or music perception (Doelling and Poeppel, 2015). However, contrary to its attractive theoretical background, studies investigating neural entrainment are often criticized, due to an apparent failure to distinguish such *predictive oscillatory processes* from other neural activity that can potentially produce similar data, or due to a lack of understanding of the underlying neural mechanisms.

**Figure 1:**
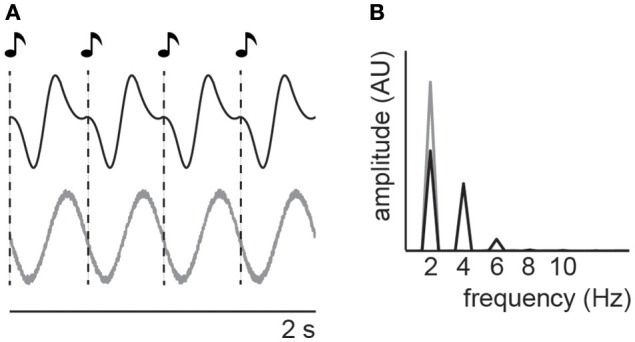
It has often been reported that neural oscillations can align to a rhythmic stimulus (shown schematically in **A**, bottom). The spectrum of this stimulus-aligned signal will reflect the dominant frequency of the stimulus (**B**, gray). However, each of the individual stimulus presentations will also evoke a neural response which, if repeated regularly, can also resemble an oscillation (shown schematically in **A**, top) and show a spectrum that reproduces the periodicity of the stimulus (**B**, black). Note that the additional peaks in the spectrum produced by the regular repetition of evoked responses reflect the imperfect sinusoidal shape of the signal which can introduce harmonic peaks in the spectrum. However, oscillations as measured with electrophysiological methods are often far from perfect sinusoids (Cole and Voytek, 2017), potentially increasing the similarity between aligned oscillations and regularly occurring evoked neural responses, and excluding harmonics in the spectrum of the signal as a criterion to distinguish the two.

Maybe the most critical issue is the relatively trivial observation that by presenting a stimulus that fluctuates at a certain frequency, we can measure a brain response at the same frequency. This phenomenon has been described long ago (Adrian and Matthews, 1934) and termed steady-state evoked potentials (Chatrian et al., 1960; Regan, 1966). More precisely, if we present a given stimulus N times per second (e.g., four syllables per second for a typical snippet of speech), it is unsurprising that we observe N neural responses per second, and the superposition of these responses might look like an oscillatory signal that is aligned to the stimulus rhythm (Figure 1), without necessarily involving generators of *endogenous oscillatory activity*. Indeed, is has been suggested repeatedly that what is commonly termed “neural entrainment” is nothing else than a regular repetition of evoked neural potentials (Capilla et al., 2011; Keitel et al., 2014)—a conclusion with tremendous consequences for theories that are critically based on entrainment. Despite this criticism, large parts of the current literature implicitly or explicitly assume that (1) an alignment between recorded neural signal and stimulus rhythm necessarily involves endogenous oscillatory activity and that (2) this stimulus-aligned oscillatory activity includes, or is even synonymous with, predictive processes.

In this article, we try to take a step back and review evidence for these assumptions that are, although sometimes debated (Capilla et al., 2011; Keitel et al., 2014; Zoefel and VanRullen, 2015b; Breska and Deouell, 2017), most often accepted without being thoroughly challenged. We start from the observation that the presentation of a stimulus that fluctuates at a given frequency often produces a brain response that fluctuates at the same frequency (Figure 1A)—this is the steady-state evoked potential described above. Here, we only cover a subset of this vast research field, as explained below: We do not question the fact that this rhythmic brain response entails a regular repetition of evoked neural responses; on the contrary, a presented stimulus will always evoke a purely sensory-driven response. We rather ask, does the measured signal *additionally* reflect endogenous oscillatory activity? Note that we use the term “endogenous oscillatory activity” synonymously with “neural entrainment” if it is aligned with rhythmic input, but we prefer the former, as it makes assumptions about the underlying neural mechanisms explicit. The scope of this review is not to summarize the state-of-the-art in entrainment (or steady-state evoked response) research, but rather to focus on studies that provide evidence on how neural oscillations and evoked neural responses can be disentangled. We summarize several studies that, using complementary state-of-the-art methods and theoretical approaches, build the fundament of our conclusion that, although sparse, there is increasing evidence for endogenous oscillatory activity being involved in brain responses to rhythmic stimulus input. We group studies into categories (see also Table 1): Each of them is based on a theoretical argument of how endogenous oscillatory activity can be dissociated from evoked neural responses and underlined with experimental evidence. We point out remaining issues that need to be clarified in future studies to make this evidence even more conclusive. Finally, we reflect on the often assumed (but hardly tested) functional role of stimulus-aligned oscillatory activity: A predictive amplification or attenuation of expected stimulus input (e.g., Schroeder and Lakatos, 2009; Lakatos et al., 2013), i.e., predictive oscillatory processes. Note again, that this review is not a comprehensive overview of neural mechanisms underlying prediction, but rather summarize evidence that stimulus-aligned (“entrained”) neural oscillations reflect an anticipation of upcoming stimulus input, as commonly assumed. Whereas endogenous oscillatory activity is a prerequisite for predictive oscillatory processes, not all oscillations must reflect predictions—thus, whenever evidence in favor of endogenous oscillatory activity can also be seen as a supporting argument for predictive oscillatory processes, this is mentioned explicitly.

**Table 1:** Summary of evidence for endogenous oscillatory activity (EOA) and predictive oscillatory processes (POP) involved in the processing of rhythmic stimulus input.

**Study**	**Evidence for EOA**	**Add. Evidence for POP**	**Remaining issues**
Nozaradan et al., 2011, 2012; Celma-Miralles et al., 2016; Tal et al., 2017	Brain responses “track” perceived beat and internally generated rhythms.	No	Do imaginary rhythms evoke neural responses?
Ding et al., 2016; Makov et al., 2017	Brain responses “track” linguistic structure not reflected in stimulus spectrum.	No	Does the extraction of linguistic structure evoke neural responses?
ten Oever et al., 2017	Phase alignment to rhythm below detection threshold.	Yes	How do subthreshold stimuli influence oscillations without evoking neural responses?
Zoefel and Heil, 2013	Oscillatory response to undetected rhythmic tone sequences.	Yes	How do subthreshold stimuli influence oscillations without evoking neural responses?
Zoefel and VanRullen, 2015a, 2016; Zoefel et al., 2017	Brain responses “track” speech rhythm without slow systematic fluctuations in spectral energy.	No	Do “high-level” features of speech evoke neural responses?
Halbleib et al., 2012; Mathewson et al., 2012; Lakatos et al., 2013; Spaak et al., 2014	Oscillatory activity after the offset of a rhythmic stimulus.	Yes	Can the aftereffect be explained by “filter ringing”?
O'Connell et al., 2011, 2014; Lakatos et al., 2013	Neural alignment to stimulus rhythm in brain regions and cortical layers in which evoked neural responses are weak or absent.	No	
Luo et al., 2013; ten Oever et al., 2017	Phase alignment in the absence of power effects, or decrease of power.	No	Stimulation frequency different from neural frequency in Luo et al. (2013) Power analyses less powerful than phase analyses.
Kayser et al., 2015	Jitter in stimulus rhythm has consequences on brain responses that are different from those expected for evoked neural responses.	Yes	Opposing findings in Capilla et al. (2011)
Notbohm et al., 2016	Brain responses to rhythmic (visual) stimuli can be characterized by an “Arnold Tongue”.	No	
Zaehle et al., 2010; Helfrich et al., 2014; Minami and Amano, 2017	tACS affects neural activity as expected for endogenous rhythms.	No	Can tACS effects be compared with rhythmic sensory stimulation?
Herring et al., 2015; Kizuk and Mathewson, 2017	Impact of attention on brain response to rhythmic stimulus or TMS is different from that expected for evoked neural responses.	No Yes	Results need to be reconciled with studies reporting different attentional effects on brain-stimulus alignment.
Notbohm and Herrmann, 2016	Modulation of visual detection depends on whether stimulation “history” is rhythmic or irregular.	No	
Mathewson et al., 2012; de Graaf et al., 2013; Hickok et al., 2015; ten Oever and Sack, 2015	Periodic modulation of behavior after offset of rhythmic stimulus.	Yes	Can the aftereffects be explained by “filter ringing”?

## Evidence from paradigms avoiding measurable evoked neural responses

While some of the arguments presented in this paper can be considered a sufficient argument for endogenous oscillatory activity—in as such they can reject explanations based on evoked responses—none of them is a necessary argument as we often expect brain responses to a rhythmic stimulus to entail a mixture of both, evoked responses and oscillatory activity. For example, finding a strong evoked response does not necessarily lead to the absence of endogenous oscillatory activity. However, the fact that endogenous oscillatory activity and regular evoked potentials share several signal properties (e.g., the dominant frequency in response to a rhythmic stimulus) does make it difficult to dissociate endogenous oscillatory activity from evoked responses if they are both present in the recorded (electrophysiological) signal. Hence, the arguably most straightforward and easiest way to distinguish endogenous oscillatory activity from regular evoked responses is a paradigm in which the former can be detected while no measurable evoked responses are produced. This could be the “tracking” of a rhythm that is created *by* the brain and therefore not present in the input (such as an imaginary beat), or to a stimulus that is too weak to create measurable evoked neural responses. Indeed, some studies have demonstrated entrainment in such paradigms, as described in the following.

### Brain responses to internally constructed rhythms

In a series of experiments, Nozaradan et al. (2011, 2012, 2015) and Nozaradan (2014) presented auditory beats to their participants and measured neural responses using electroencephalography (EEG). Not surprisingly, they reported dominant frequencies in the spectrum of the neural signal that closely followed the spectrum of the acoustic stimulus (see also Henry et al., 2017; Nozaradan et al., 2017). However, more striking is their observation that this is not always the case: Not only did they find a selective enhancement of frequencies related to the perceived beat and meter (Nozaradan et al., 2012), but they also reported neural responses at frequencies corresponding to an imaginary rhythm (Nozaradan et al., 2011), a finding that was later confirmed for the visual domain (Celma-Miralles et al., 2016). Similarly, Tal et al. (2017) showed that neural activity can “track” a perceived rhythm induced by a pattern of drum beats even though the frequency of this rhythm is not visible in the spectrum of the stimulus. These findings exclude bottom-up evoked responses as an explanation, as the rhythm was not physically present in the stimulus, but instead produced internally by the participant (i.e., the beat).

**Figure 2:**
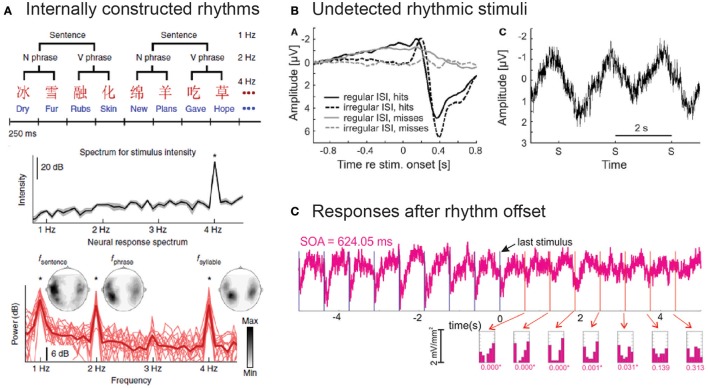
Overview of selected studies that show endogenous oscillatory activity in the absence of measurable evoked responses. **(A)** Ding et al. (2016) constructed speech sentences in which not only words (4 Hz) but also phrases (2 Hz) and sentences (1 Hz) fluctuated rhythmically (top). The spectrum of these stimuli only reflected the word rate, but not the rhythm of phrases or sentences (middle). Apart from brain responses at the frequency of words, the authors also observed neural activity fluctuating at phrase and sentence rates, even though these were not present in the stimulus spectrum (bottom). Asterisks mark frequency bins with significantly higher power than neighboring bins (see Ding et al., 2016, for details). Reproduced with permission from Ding et al. (2016). **(B)** In the study by Zoefel and Heil (2013), only detected but not undetected auditory stimuli produced a noticeable evoked neural response (left; no stimuli were presented during catch trials). Nevertheless, brain activity averaged across instances of three subsequently missed stimuli revealed an oscillatory pattern at stimulation frequency (right; S denotes the timing of stimulus presentation). Reproduced with permission from Zoefel and Heil (2013). **(C)** Lakatos et al. (2013) recorded neural activity in monkey auditory cortex during and after the presentation of rhythmic auditory stimuli. They found that, even after the offset of the rhythmic stimulus sequence, the neural phase at the time of expected stimulus occurrence (shown by vertical lines) is strongly biased toward a particular phase (see insets for a phase distribution across trials). This phenomenon was visible both in tonotopical regions tuned to the sound frequency of the stimulus (shown) and in those who are not (not shown). Reproduced with permission from Lakatos et al. (2013).

A very similar approach and finding was presented by Ding et al. (2016), using speech as the entraining stimulus. In a clever paradigm, they constructed speech sounds (Figure 2A) with regular fluctuations at the word (4 Hz), phrase (2 Hz), and sentence level (1 Hz); importantly, only the fluctuations at the word rate were reflected in the spectrum of the acoustic stimulus, as both phrases and sentences are rather a logical construct imposed by the listener than a consequence of the physical stimulus properties. Strikingly, Ding et al. (2016) reported peaks in the neural spectrum that corresponded to the rhythm of phrases and sentences, and these peaks were only present for native speakers of the presented language, Chinese (Figure 2A). These findings were replicated by Makov et al. (2017); they could also show that the “tracking” of these “high-level” structures (phrases and sentences) was abolished during sleep. Together, these results suggest that rhythmic fluctuations in endogenous oscillatory activity can be observed in scenarios in which we would not expect steady-state responses to fluctuations in the stimulus.

### Brain responses to subthreshold or undetected stimuli

An alignment of endogenous oscillatory activity to subthreshold stimuli was demonstrated by ten Oever et al. (2017). In this study, auditory stimuli were either presented at a rhythmic presentation rate of 1.5 Hz or using random inter-stimulus intervals, maintaining an average rate of 1/1.5 s. Using magnetoencephalography (MEG) and electrocorticography (ECoG), the authors quantified evoked neural responses based on a well-known component of event-related potentials (N100m) and the assumption that a regular repetition of evoked responses would result in a change of power in the recorded signal at the frequency of stimulus presentation (see also section Power Changes vs. Phase Alignment). The alignment between electrophysiological signal and stimulus rhythm was measured using inter-trial coherence (i.e., the phase consistency of the recorded signal across trials). Interestingly, results revealed that evoked neural responses were only present for successfully detected stimuli. At the same time, inter-trial coherence was enhanced for the rhythmic, but not for the random presentation rate for stimuli that were presented below perceptual detection thresholds, i.e., remained undetected. Moreover, the inter-trial coherence pattern for the subthreshold 1.5 Hz oscillation was confined to a narrow (1.5 Hz) frequency range, while evoked responses are expected to affect the spectrum of the recorded signal in a wider band (see e.g., Will and Berg, 2007). Thus, this study provides evidence for endogenous oscillatory activity aligned to the stimulus rhythm. It also represents support for predictive oscillatory processes, as thresholds for detecting rhythmic stimuli were lower than for random stimuli, suggesting that the rhythmic alignment supports perception (see also ten Oever et al., 2014).

Zoefel and Heil (2013) presented listeners with auditory stimuli at a rate of 0.5 Hz. These sounds were presented at near-threshold level so that only half of them were actually perceived by the participants; importantly, undetected stimuli did not produce a distinct evoked response (Figure 2B). Critically, a pronounced oscillation at 0.5 Hz was visible even for an interval of three subsequent misses, demonstrating an involvement of endogenous oscillatory activity in the processing of these stimuli (Figure 2B). This finding might also represent predictive processes as the functional role behind stimulus-aligned oscillatory activity, as the latter was present and adapted to the expected occurrence of the stimulus even if the latter remained undetected, potentially reflecting a mechanism of temporal prediction.

### Brain responses in the absence of slow spectral energy fluctuations in the stimulus

Endogenous oscillatory activity is particularly prominent in current theories of speech processing (Giraud and Poeppel, 2012; Peelle and Davis, 2012; Ghitza, 2013). It is all the more important to emphasize that everyday speech consists of large amplitude fluctuations, and the apparent oscillatory activity might merely reflect a passive “following” of these fluctuations in the stimulus. Zoefel and VanRullen (2015a) therefore reported the construction of a novel speech/noise stimulus in which these slow fluctuations in spectral energy have been removed. In a series of experiments, they demonstrated that oscillatory activity persists in the absence of pronounced spectral energy fluctuations: The perception of embedded auditory targets (Zoefel and VanRullen, 2015a), EEG oscillations (Zoefel and VanRullen, 2016), and intracortical oscillations in monkey A1 (Zoefel et al., 2017) aligned with the rhythm of the remaining speech features (summarized in Zoefel and VanRullen, 2015b); these results support the idea that the processing of speech does include endogenous oscillatory activity and is not restricted to a “following” of amplitude fluctuations in the stimulus.

### Brain responses after the offset of a rhythmic stimulus

Another important line of evidence comes from the repeatedly reported observation that endogenous oscillatory activity can be observed *after* the offset of a rhythmic stimulus. Logically, evoked responses can be ruled out in the absence of a stimulus, so that any oscillatory behavior of neural signals can be assigned to endogenous oscillatory activity induced by the preceding stimulus rhythm. Indeed, in different modalities, electrophysiological signals have been shown to oscillate after stimulus offset (Figure 2C; in audition: Lakatos et al., 2013; and vision: Halbleib et al., 2012; Mathewson et al., 2012; Spaak et al., 2014; but see the negative finding reported by Capilla et al., 2011). Importantly, these findings represent crucial evidence of predictive oscillatory processesas the underlying functional mechanism: endogenous oscillatory activity is not only aligned with actual stimulus onset, but with the *expected* occurrence of upcoming stimuli, in support for the notion that the alignment between endogenous oscillatory activity and rhythmic stimulus input can reflect a mechanism of temporal prediction (Schroeder and Lakatos, 2009).

### Layer- and stimulus-specific brain responses

In recent years, intracranial recordings (in particular in primate sensory cortices) have contributed critically to our knowledge of the neural mechanisms underlying rhythmic stimulus processing. An important advantage of these methods is the possibility to record neural activity from well-defined cortical locations, with respect to stimulus tuning (e.g., tonotopy in the case of auditory processing) and cortical layers.

Some of these insights can be used to differentiate endogenous oscillatory activity from evoked neural responses. Specifically, endogenous oscillatory activity is thought to be organized through both local lateral connections (Llinás, 1988; Somers and Kopell, 1993; Buzsáki and Draguhn, 2004) as well as long-range connections, e.g., the cortico-thalamic loop (Llinás, 1988; Steriade et al., 1990). Both local lateral and thalamic-cortical connections mostly involve supra- and/or infragranular layers; endogenous oscillatory activity can therefore be expected to be strongest in these (as compared to granular) layers (see e.g., Bollimunta et al., 2008; Haegens et al., 2015). Indeed, an alignment between neural activity and stimulus rhythm is most frequently observed in supra- or infragranular layers whereas evoked activity is strongest in granular layers (Lakatos et al., 2005, 2008, 2013; O'Connell et al., 2011, 2014). Also, in the auditory domain, the synchronization between brain signals and rhythmic sounds can be observed in areas of A1 not tuned to the presented sound frequency (e.g., two or more octaves away from the “best” frequency, i.e., the sound frequency that evokes the strongest neural response in that area) and therefore evoke only very weak or no neural responses (O'Connell et al., 2011; Lakatos et al., 2013). Both observations are thus difficult to explain based on a regular repetition of evoked neural responses and are strong evidence for endogenous oscillatory activity aligned with the stimulus rhythm. Finally, as mentioned above, stimulus-aligned neural oscillations are thought of as a mechanism to optimize processing of future stimulus events (Schroeder and Lakatos, 2009), by definition a top-down process. It has been suggested that top-down processes mostly affect neural activity in the supra- and infragranular layers of the sensory cortices (Maunsell and van Essen, 1983). As reported in this section, the strongest alignment of brain signals to rhythmic stimulation (but not stimulus-evoked responses) can be found in these layers. Thus, these findings do not only support the idea of an involvement of endogenous oscillatory activity but also underline its potential functional role as a predictive mechanism.

### Paradigms avoiding measurable evoked neural responses: unresolved issues

Although the evidence for endogenous oscillatory activity in response to rhythmic stimulation reported here is promising, some issues remain that need to be addressed in future studies: For instance, can endogenous oscillatory activity at a frequency that is not present in the spectrum of the rhythmic stimulus (Figure 2A; Nozaradan et al., 2011; Ding et al., 2016; Makov et al., 2017; Tal et al., 2017) be used to argue that a signal measured in response to a stimulus that *does include* this frequency in its spectrum entails endogenous oscillatory activity as well? Moreover, any response produced by the brain must also involve neural activity of some kind. Therefore, both the imagination of a beat (Nozaradan et al., 2011; Tal et al., 2017), the extraction of structure from a series of words (Ding et al., 2016), or the processing of speech features other than spectral energy (Zoefel and VanRullen, 2015a) might also have produced evoked neural responses (see, for instance, Suess and Abdel Rahman, 2015; Mitchell and Cusack, 2016, for neural responses measured during imagery, or Krumbholz et al., 2003, for responses to sounds without energy fluctuations). These responses will appear regularly, due to the experimental design—and a contamination of the apparent oscillations by these regularly appearing neural responses cannot be ruled out. Similarly, if a sub-threshold stimulus can modulate oscillations (ten Oever et al., 2017), it influences neural activity by definition. Thus, it needs to be clarified why it is possible to influence oscillations without evoking any neural response otherwise (i.e., why the threshold for endogenous oscillatory activity is lower than for a typical evoked response).

Finally, the sinusoidal modulation of electrophysiological signals after a rhythmic stimulus is relatively short (mostly < 1 s; Figure 2C). In addition, several studies have reported oscillatory brain responses after the offset of a visual stimulus flickering at a frequency that does not necessarily correspond to a typical endogenous oscillation (e.g., 24 Hz in Keitel et al., 2014; see also Clementz et al., 2004). Thus, it needs to be tested whether the reported aftereffects can be explained by the “ringing” of a simple “brain filter” involved in stimulus processing, potentially by modeling such a filter and how (long) it would affect the measured response.

## Evidence from signal properties that are expected to differ between endogenous oscillatory activity and regular evoked potentials

Although endogenous oscillatory activity and regular evoked potentials share different signal properties, there are also some characteristics that we would *only* expect to find in electrophysiological data if it were produced by endogenous oscillatory activity—but not by evoked potentials. These signal properties that are *specific* for endogenous oscillatory activity build the second category of arguments presented in this paper and are described in detail below.

### Power changes vs. phase alignment

Previous literature often focused on the impact of stimulation parameters (e.g., stimulus frequency or transient events) or cognitive variables (e.g., attention) on the phase or amplitude of steady-state evoked potentials (e.g., Morgan et al., 1996; Ding et al., 2006; Kim et al., 2007; Moratti et al., 2007). However, based on this approach, it is difficult to draw conclusions on the involvement of endogenous oscillatory activity: For instance, an attended stimulus produces a larger evoked neural response as compared to an unattended one (Haider et al., 1964). In this section, we focus on rather theoretical considerations of the changes (e.g., as compared to an interval without stimulation) we would expect in a brain response to a rhythmic stimulus if it were produced by regular evoked neural responses versus stimulus-aligned neural oscillations.

Assuming that evoked responses, as they are typically measured with electrophysiological methods such as EEG/MEG, reflect neural activity evoked by the stimulus *in addition* to spontaneous or “background” neural activity, it is a logical consequence that regular evoked responses should produce an increase in power in the spectrum of the recorded signal at the stimulation frequency (and potentially other frequencies). This does not have to be the case for endogenous oscillatory activity which could simply entail the alignment of the oscillatory phase to the external rhythm (Shah et al., 2004; Sauseng et al., 2007). In particular if the stimulation frequency matches the frequency of a natural ongoing oscillation, one would not necessarily expect an increase in power at this frequency, but rather an increase in phase-locking between recorded signal and presented stimulus that develops over time (e.g., Thut et al., 2011). For example, Luo et al. (2013) presented three concatenated random noise sequences to participants, each 0.5 s long. Unknown to the participants, one particular sequence was repeated more often than others. The authors found that MEG phase at relatively low frequencies (3–8 Hz) was more consistent during the presentation of the repeated stimulus as compared to non-repeated sequences. This phase consistency in the MEG signal developed over trials while participants implicitly learned the sequences, suggesting a functional role. Moreover, the observation was not paralleled by an increase in neural power, suggesting an involvement of endogenous oscillatory activity, in line with the argumentation in this section. However, it is noteworthy that it is difficult to claim that endogenous oscillatory activity was indeed aligned with the rhythm of the stimulus, as the dominant frequencies in the stimulus and neural spectrum were not identical.

It is likely that the alignment of endogenous oscillatory activity with a rhythmic stimulus involves a phase-reset of neural oscillations in order to align their phase with the rhythmic input (Lakatos et al., 2009). Typical time-frequency analyses include multiple cycles of an oscillation, but assume stationarity within the analysis window. This assumption is necessarily violated if a phase-reset occurs, since it will change the ongoing phase of the oscillation. As a consequence, a sinusoidal signal fitted to the data (e.g., during FFT) will not explain as much variance of the signal as for an ongoing oscillation without stimulus input (i.e., phase-reset). This leads to a decreased power (as compared to a pre-stimulus baseline) in the output of the analyses while entrainment is not yet complete (i.e., while the phase relation between oscillation and stimulus is not yet stable). Note that a response evoked by a non-rhythmic stimulus (i.e., without the involvement of oscillatory activity) can be phase-consistent across trials as well, leading to a similar phenomenon. However, evoked neural responses can be assumed to include *additional* brain activity (in contrast to phase-reset endogenous oscillations), leading to an increased power in the signal and compensating for the potential power decrease described above. Thus, a decrease in signal power at the frequency of interest would be an indication for the involvement of endogenous oscillatory activity.

**Figure 3:**
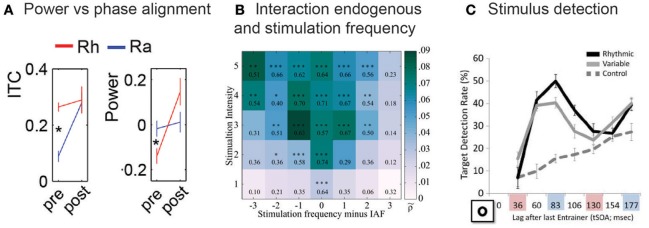
Overview of selected studies providing evidence from signal properties and cognitive effects that are expected to differ between endogenous oscillatory activity and regular evoked responses. **(A)** ten Oever et al. (2017) reported higher inter-trial coherence (ITC) for rhythmic (Rh) compared random (Ra) auditory subthreshold stimuli stream (sub-threshold trials are denoted “pre”; supra-threshold trials are denoted “post”). The higher ITC was paralleled with lower power values for the rhythmic compared to the random subthreshold sounds. Asterisks indicate a significant difference (*p* < 0.05) between rhythmic and random conditions. **(B)** The intensity required for a rhythmic stimulus to “entrain” an endogenous oscillation depends on the distance between the stimulus frequency and the natural frequency of the oscillator. This relationship results in a triangular shape representing the dependence of entrainment strength on frequency and intensity of the entraining stimulus, the so-called Arnold Tongue. Notbohm et al. (2016) reported that the EEG signal in response to a rhythmic visual stimulus follows the characteristics of an Arnold Tongue, if it is analyzed based on participants' individual alpha frequency (IAF). Reproduced with permission from Notbohm et al. (2016). **(C)** Mathewson et al. (2012) measured the probability of detecting a visual target at different time lags after the offset of a rhythmic visual stimulus (~12 Hz). They found a periodic modulation of perception (i.e., target detection); these aftereffects were also present when a certain jitter was introduced in the rhythm, but not after the presentation of two visual stimuli separated by a certain time interval (576 ms) but without any rhythmic component (control). Reproduced with permission from Mathewson et al. (2012).

Most entrainment studies focus on measures of phase alignment instead of power analyses, thereby potentially missing an important piece of evidence that could differentiate evoked responses from endogenous oscillatory activity. So far reports of a power decrease during rhythmic stimulation are limited. As an example, in the study by ten Oever et al. (2017), described above, a significantly higher power at a frequency corresponding to the presentation rate of random as compared to rhythmic undetected auditory sequences was found (Figure 3A), but only in ECoG and not in MEG responses. More evidence is needed to validate that this decrease in power can be observed reliably. In particular, it is difficult to draw conclusions from an absence of a power increase as we are inferring something from a possible type I error. Power analyses are shown to be less powerful than analyses of phase-locking (Ding and Simon, 2013). Thus, it is important to ensure high statistical power in experimental settings. Moreover, power and phase measurements are not fully independent from each other, since an increase of power can improve the reliability of any phase estimate. The shape of neural oscillations as commonly observed in electrophysiological recordings is also far from perfectly sinusoidal (Cole and Voytek, 2017); this can introduce peaks at harmonic frequencies in the signal's power spectrum (Figure 1B) and calls for further caution when using power effects as critical support for a hypothesis. Finally, it is important to stress that an increase in power does not equate the absence of endogenous oscillatory activity, as it can also be seen as a mechanism initiated by the brain to enhance predictive oscillatory processes: For instance, an increase in power might increase the efficacy of oscillatory phase, and therefore increase the impact of high-excitability (i.e., stimulus amplification) or low-excitability (i.e., stimulus attenuation) phase, and its usefulness for the brain to “gate” expected input.

### Brain responses to jitter in the input rhythm

Several studies have shown that quasi-rhythmic input (i.e., a rhythmic stimulus with slightly jittered inter-stimulus intervals) can lead to frequency-selective brain responses (i.e., corresponding to the applied frequency range; e.g., Stefanics et al., 2010; Cravo et al., 2013; Keitel et al., 2017). Whereas endogenous oscillatory activity and a superposition of regular evoked responses might look very similar if the stimulus is perfectly periodic, we would expect differences if a jitter is introduced in the input rhythm. Specifically, assuming that stimulus-aligned oscillatory activity reflects predictive processes, the onset time of the upcoming stimuli might be proactively monitored and responses adapted according to changes in the stimulation pattern. This might lead to an oscillatory signal at a frequency corresponding to the *average* presentation rate of the stimulus, or to a constant adaptation of the oscillatory frequency depending on the preceding N inter-stimulus intervals (see Capilla et al., 2011). The frequency of the oscillation might even be adapted based on the expected occurrence of the *next* event if the latter is predictable despite the introduced jitter (speech might be such an example, as the onset of a given word might be predictable based on preceding words despite speech not being perfectly periodic). In either case, a similar behavior would be unlikely for regular evoked responses which would be expected to passively follow the timing of presented stimuli without proactive modulation.

Based on this idea, Capilla et al. (2011) compared EEG signals, recorded in response to jittered visual input, with two different models: One dataset modeled to be produced by evoked responses and another reflecting oscillatory responses whose instantaneous frequency depended on the preceding inter-stimulus intervals (i.e., including predictive oscillatory processes). Specifically, they hypothesized that the timing between two major components of neural responses to a stimulus (N75 and P100) would only depend on the preceding inter-stimulus intervals (e.g., longer latencies between N75 and P100 for longer preceding intervals) if endogenous oscillatory activity (which should be continuously adapted to changes in these intervals, i.e., to changes in the instantaneous presentation rate) underlies the observed responses. Importantly, they found that this is not the case; the experimental data could thus be best explained by a superposition of evoked responses. However, an opposite conclusion was made in the work by Kayser et al. (2015). In their study, EEG was recorded while participants listened to speech sounds manipulated by changing the timing of silent gaps between words or syllables (i.e., the speech was made more irregular, while maintaining a constant average inter-stimulus interval). They reported that the introduced jitter did not affect brain responses to transients in the stimulus. At the same time, however, the mutual information between delta oscillations (0.5–2 Hz) and speech envelope decreased with increasing jitter, suggesting that endogenous oscillatory activity and evoked neural responses can be dissociated. It would be interesting to test whether the system is specifically “tuned” to the characteristics of everyday speech so that mutual information between EEG and speech envelope decreases in response to more irregular, but also more regular speech. As a cautionary note, however, even though the average brain response to transients in the signal was similar across different jitter conditions, the *repetition* of these individual responses followed, by construction, the introduced jitter. A more jittered stimulus would therefore also result in more jittered brain responses, with unknown effects on the quantification of mutual information.

As suggested in this section, modeling neural activity in response to jitter in the stimulus rhythm is a promising way to disentangle evoked neural responses from endogenous oscillatory activity (see also Breska and Deouell, 2017; Obleser et al., 2017). However, there are still several challenges that need to be addressed in adequate models. For example, the predictive nature of endogenous oscillatory activity has to be characterized in more detail: How do oscillations “track” the input, i.e., based on global or local temporal statistics, or both? Do they interact with evoked responses and, if yes, in which manner? These and other issues need to be investigated in future studies.

### Interaction Between endogenous frequency, stimulation frequency, and stimulation intensity

Further evidence for endogenous oscillatory activity in response to rhythmic stimulus input stems from more theoretical considerations that have been underlined with experimental data. If an oscillator is coupled (i.e., entrained) to an external rhythm, it shows a behavior that critically depends on the interaction between its endogenous frequency and both frequency and intensity of the stimulation. Importantly, this leads to certain characteristics in how neural oscillations respond to rhythmic input that can be assumed to be specific for endogenous oscillatory activity, i.e., not present for a regular repetition of evoked neural responses.

For instance, a classical physical model predicts that entrainment effects are strongest when two oscillators are matched in their frequency (Pikovsky, 2008)—and therefore, for stimulation rhythms that match the frequency of the endogenous oscillation (Ali et al., 2013; Fröhlich, 2015). Beyond a perfect match, the degree of alignment between endogenous oscillatory activity and stimulus would depend both on the intensity of the rhythmic stimulus and the distance between the frequency of the latter and the eigenfrequency of the oscillator, resulting in the so-called Arnold Tongue (Figure 3B; Pikovsky, 2008; Ali et al., 2013; Fröhlich, 2015). Moreover, at the border of the Arnold Tongue, intermittency can be expected, reflected by an irregular alternation of phase synchronization and decoupling between oscillator and stimulus. Indeed, Notbohm et al. (2016) showed that both properties can be observed in EEG data recorded in response to regular visual stimulation: Not only did the synchronization of EEG oscillations to the visual flicker depend on both stimulation intensity and the distance between flicker frequency and the participants' individual alpha frequency (IAF; Figure 3B), they also observed intermittency at the border of the Arnold Tongue. Importantly, outside the Arnold Tongue, they found that the behavior of EEG responses to regular flicker did not differ from that in response to jittered stimulation (e.g., responses did not depend on the distance to the IAF anymore), indicating a superposition of evoked neural responses if the frequency of the stimulus is too far away from that of an endogenous oscillator.

In a similar vein, effects of transcranial alternating current stimulation (tACS) have most frequently been reported in the alpha range (~8–12 Hz), the most prominent EEG rhythm of the brain (Zaehle et al., 2010; Helfrich et al., 2014). Specifically, tACS can increase the participants' EEG power at the corresponding frequency if applied at alpha, but not at other frequencies (Zaehle et al., 2010; Helfrich et al., 2014), an effect that can last up to 60 min after stimulation (reviewed in Strüber et al., 2015). It also seems possible to use tACS to shift the participants' individual alpha frequency toward the tACS frequency (Helfrich et al., 2014; Minami and Amano, 2017), but it is unclear if and how long the effect lasts when tACS is turned off (Minami and Amano, 2017). Nevertheless, these findings argue against neural activity merely “following” the applied current as (1) the stimulation seems to be particularly effective for frequencies matching endogenous rhythms, (2) stimulation effects can last longer than the stimulation itself and (3) a *shift* in the dominant neural frequency, but not an additional peak at stimulation frequency, has been observed in the spectrum. While entrainment research with non-invasive brain stimulation is informative for extracting endogenous oscillatory activity, it is unclear whether the entrainment exhibited after the stimulation of neuronal populations in sensory cortices leads to the same entrainment phenomena found during rhythmic sensory input (Vossen et al., 2015). Nevertheless, it shows us that neural mechanisms exist that can adjust oscillatory activity to the characteristics of the input the system receives.

Another important, but rarely studied, issue is that the eigenfrequency of an endogenous oscillations does not have to be constant, but might be flexible to a certain degree (see also section Brain Responses to Jitter in the Input Rhythm). For instance, the individual alpha frequency depends on luminance (Benedetto et al., 2017), and theta oscillations (~4–8 Hz), assumed to be critical for speech perception, necessarily have to adapt their frequency in order to track the continuous changes in the rhythm of a typical sentence (e.g., Ghitza, 2011). Indeed, speech perception is disrupted if the frequency of speech rhythm exceeds the range these oscillations presumably can operate in (Ghitza, 2014). In the motor domain, participants can tap to a variety of metronome frequencies, but seem most accurate at their own preferred tapping pace (Collyer et al., 1994; Repp, 2005). According to the theoretical model of the Arnold Tongue described above, given a sufficiently high stimulus intensity, an endogenous oscillator can be entrained even if its “preferred” frequency does not match that of the stimulus. However, oscillatory mechanisms of stimulus processing might be most relevant at lower intensity levels (i.e., near detection threshold), where the neural high-excitability phase can “boost” the stimulus above detection threshold, whereas the low-excitability phase can prevent stimulus detection (e.g., Busch et al., 2009). Thus, even though a supra-threshold stimulus might therefore be more “powerful” in entraining oscillatory activity, the system might operate at perceptual limits where oscillatory mechanisms are irrelevant—at least for perception. This assumption indicates that a mere increase of stimulus intensity is not an adequate alternative for a mismatch between stimulus and endogenous frequency. The issues described here need to be implemented in updated models describing the relationship between endogenous oscillations, stimulation frequency, and intensity.

## Evidence from cognitive effects that are expected to differ between endogenous oscillatory activity and regular evoked potentials

Not only some properties of electrophysiological signals can be expected to differ, depending on whether they reflect endogenous oscillatory activity or evoked neural responses, but also the impact of cognitive processes on the observed brain response, and the consequences for perception and behavior. Some of these differences are straightforward—for instance, periodic fluctuations in perception after the offset of the rhythmic stimulus would only be expected as a consequence of endogenous oscillatory activity—others are more subtle to detect. In recent years, clever paradigms have been developed in which endogenous oscillatory activity and regular evoked responses can indeed be disentangled. These studies are summarized in this section.

### Effect of attention

Herring et al. (2015) designed a study in which they measured the effect of attention on alpha oscillations produced by a transcranial magnetic stimulation (TMS) pulse. The question underlying their experiment was relevant to the present article: Can a TMS pulse modulate neural oscillations or does it only produce a rhythmic burst of evoked neural activity? To differentiate these alternatives, they tested how attention affects the EEG measured after a TMS pulse. They found that attention suppressed TMS-induced oscillatory activity at 10 Hz; critically, this behavior would only be expected for endogenous oscillatory activity whereas the opposite (i.e., an increase) would represent a typical attentional effect for evoked neural responses. It should be noted here that there was no rhythmic input in this study, as only a single TMS pulse was applied. Nevertheless, although the single pulse did not produce any temporally predictive value, the reported findings represent evidence that it can *reset* neural oscillations—as mentioned above, an important mechanism that might be necessary for the alignment of endogenous oscillatory activity to a rhythmic stimulus. Repeated TMS (rTMS), commonly used to study entrainment (Thut et al., 2011), might therefore also influence endogenous oscillations. Maybe even more important, the experimental design presented by Herring et al. opens up new possibilities for entrainment studies: If attention is expected to affect endogenous oscillatory activity and evoked responses in different ways, the two can be disentangled.

Indeed, in a recent study, Kizuk and Mathewson (2017) demonstrated that a lack of visual attention does not only increase alpha power, but also the effects of an rhythmic stimulus (in this case, at 12 Hz) on neural activity and behavior that can be observed after the offset of the stimulus (see section Brain Responses after the Offset of a Rhythmic Stimulus and Effect on Stimulus Detection). Nevertheless, it has been described repeatedly that attention *increases* the alignment of endogenous oscillatory activity to a stimulus, at least at frequencies below the alpha band (Stefanics et al., 2010; Lakatos et al., 2013). It needs to be clarified in future studies how these findings can be reconciled, whether the reported attentional effects are frequency-dependent, or if results differ depending on whether effects of attention are tested during or after the rhythmic input. Together with results described in section Interaction between Endogenous Frequency, Stimulation Frequency, and Stimulation Intensity, these studies suggest an important interaction between endogenous oscillatory activity and rhythmic input that might be captured using clever experimental manipulations. For instance, if attention affects endogenous alpha oscillations, do we observe similar results when participants close their eyes (which affects alpha oscillations as well)? Contrary to this hypothesis, a recent study reported that tACS is more effective in entraining alpha oscillations during an eyes-open (as compared to an eyes-closed) state (Ruhnau et al., 2016). Thus, the interaction between endogenous oscillatory activity, rhythmic stimulation, and experimental parameters is a complex, but exciting field of research that merits further investigation.

### Effect on stimulus detection

Neuronal oscillations reflect rhythmic changes in the membrane potential of neuronal ensembles (Buzsáki and Draguhn, 2004), suggesting that there are phases at which stimulus processing, and therefore behavioral performance, is optimized. This is corroborated by the observation that performance in various tasks and sensory modalities depends on the phase of ongoing (“spontaneous”) neural oscillations (see VanRullen, 2016, for an elaborate review). Consequently, one might argue, if brain responses to rhythmic sensory input involve endogenous oscillations, the neural phase should influence behavior in a similar way. Several studies reported that this is indeed the case; these results are summarized in the current section.

In a study by Notbohm and Herrmann (2016), visual targets were embedded in regular or irregular visual flicker. The authors found that the modulation of target detection by the flicker differed between conditions (regular and irregular); importantly, however, the flicker sequence immediately before and after the target was identical for all conditions. Thus, the observed effect cannot be due to differences in evoked neural responses at the time of target occurrence, but rather due to stronger endogenous oscillatory activity in the case of preceding regular flicker. More studies compared rhythmic to random presentation, but not all controlled for direct local temporal statistics (Jones et al., 2002; ten Oever et al., 2014).

If the synchronization between brain response and rhythmic input indeed reflected a predictive mechanism with consequences for the processing of upcoming events, as often assumed (Large and Jones, 1999; Schroeder and Lakatos, 2009), we would expect a periodic modulation of perception even after the offset of the rhythmic stimulus, and at the frequency of the latter. Whereas these aftereffects have already been described for electrophysiological signals (section Brain Responses after the Offset of a Rhythmic Stimulus; Figure 2C), an actual modulation of behavior can arguably provide the strongest support for predictive oscillatory processes as a functional mechanism behind stimulus-aligned oscillatory activity. Indeed, several studies reported cyclic modulations of stimulus detection after the presentation of a rhythmic stimulus. Mathewson et al. (2012) found a sinusoidal modulation in visual detection rates after a visual stimulus presented at ~12 Hz (Figure 3C). This modulation was stronger for rhythmic as compared to jittered input streams. In another study, de Graaf et al. (2013) found that visual stimulus detection after both a 5.3 and 10.6 Hz stream followed (on average across participants) a sinusoidal pattern at 10.6 Hz (i.e., the first harmonic in the case of the 5.3 Hz stream). On an individual level, the frequency of the modulation of visual detection was correlated with the participants' individual alpha frequency. This result suggests that the observed effect is constrained by the frequency of endogenous oscillations and is additional evidence for an important role of endogenous oscillatory activity (see section Interaction between Endogenous Frequency, Stimulation Frequency, and Stimulation Intensity). It is an open question how long these aftereffects can last; some studies report effects that last for two or three cycles after the offset of the rhythmic input (de Graaf et al., 2013; Hickok et al., 2015; ten Oever and Sack, 2015). Together, these findings represent not only convincing evidence for brain responses to a rhythmic stimulus involving endogenous oscillatory activity but also demonstrate predictive oscillatory processes as the underlying functional mechanism.

## Conclusion

There is important and complementary evidence that endogenous oscillatory activity is involved in the response to rhythmic stimulus input. It remains to be shown how much of the observed endogenous oscillatory activity actually reflects predictive processes, as most studies demonstrating the former did not directly test the latter. Nevertheless, as the excitability fluctuations underlying endogenous oscillatory activity makes it an optimal “tool” for the “gating” of expected input (Buzsáki and Draguhn, 2004), it is a reasonable assumption that the alignment of neural oscillations can be used as the active, predictive mechanism that was originally proposed (Large and Jones, 1999; Schroeder and Lakatos, 2009). Disentangling evoked neural responses, endogenous oscillatory activity and predictive processes in future studies will be paramount for advancing this exciting field of research.

## Author contributions

All authors listed, have made substantial, direct, and intellectual contribution to the work, and approved it for publication.

### Conflict of interest statement

The authors declare that the research was conducted in the absence of any commercial or financial relationships that could be construed as a potential conflict of interest.

## Footnotes

^1^Here, we define endogenous neural oscillations as rhythmic neural activity that originates from the brain, and is therefore also present in the absence of stimulus input.
